# Design and methodology of a community-based cluster-randomized controlled trial for dietary behaviour change in rural Kerala

**DOI:** 10.3402/gha.v6i0.20993

**Published:** 2013-07-17

**Authors:** Meena Daivadanam, Rolf Wahlstrom, T.K. Sundari Ravindran, P.S. Sarma, S. Sivasankaran, K.R. Thankappan

**Affiliations:** 1 Achutha Menon Centre for Health Science Studies, Sree Chitra Tirunal Institute for Medical Sciences and Technology, Trivandrum, India; 2 Department of Public Health Sciences (Global Health), Karolinska Institutet, Stockholm, Sweden; 3 Department of Cardiology, Sree Chitra Tirunal Institute for Medical Sciences and Technology, Trivandrum, India

**Keywords:** dietary intervention, non-communicable diseases, stages of change, behavioural intervention, behaviour change, nutrition

## Abstract

**Background:**

Interventions targeting lifestyle-related risk factors and non-communicable diseases have contributed to the mainstream knowledge necessary for action. However, there are gaps in how this knowledge can be translated for practical day-to-day use in complex multicultural settings like that in India. Here, we describe the design of the Behavioural Intervention for Diet study, which was developed as a community-based intervention to change dietary behaviour among middle-income households in rural Kerala.

**Methods:**

This was a cluster-randomized controlled trial to assess the effectiveness of a sequential stage-matched intervention to bring about dietary behaviour change by targeting the procurement and consumption of five dietary components: fruits, vegetables, salt, sugar, and oil. Following a step-wise process of pairing and exclusion of outliers, six out of 22 administrative units in the northern part of Trivandrum district, Kerala state were randomly selected and allocated to intervention or control arms. Trained community volunteers carried out the data collection and intervention delivery. An innovative tool was developed to assess household readiness-to-change, and a household measurement kit and easy formulas were introduced to facilitate the practical side of behaviour change. The 1-year intervention included a household component with sequential stage-matched intervention strategies at 0, 6, and 12 months along with counselling sessions, telephonic reminders, and home visits and a community component with general awareness sessions in the intervention arm. Households in the control arm received information on recommended levels of intake of the five dietary components and general dietary information leaflets.

**Discussion:**

Formative research provided the knowledge to contextualise the design of the study in accordance with socio-cultural aspects, felt needs of the community, and the ground realities associated with existing dietary procurement, preparation, and consumption patterns. The study also addressed two key issues, namely the central role of the household as the decision unit and the long-term sustainability through the use of existing local and administrative networks and community volunteers.

Interventions targeting lifestyle-related risk factors and non-communicable diseases (NCDs) have contributed to the mainstream knowledge necessary for action. Many of these lifestyle-related risk factors are context specific (1), which becomes particularly relevant while developing interventions in complex multicultural settings like that in India. Diet, for instance, is not just about the calories consumed or the proportion of carbohydrates, proteins, and fat or adequacy of vitamins and other micronutrients in the food. It is a complex behavioural phenomenon that results from an interaction between where we live, what is available, what we can afford, how much time we have to dedicate to the process of procuring and preparing food, and even what we are allowed in terms of family traditions, societal, religious, and gender norms (2).

The link between diet and NCDs has been long established (3). In addition, other determinants have been identified as influencing dietary procurement and consumption behaviour at the individual and household (HH) level, including: (a) socio-economic status (SES) of HHs (4); (b) spending and food shopping pattern (5); (c) dietary pattern and preferences, including children's preferences (6–8); (d) overall time spent on food preparation (kitchen time) (6); (e) lifestyle changes (6); and (f) HH food culture comprising tastes, choices, beliefs, and rules related to food and meal times, structure, content, and company (9, 10). Moreover, local cultural context, political climate, economic and trade policies, and the effects of globalization all influence and shape dietary behaviours in many ways (11–13). Tackling the growing burden of NCDs related to unhealthy diet, therefore, necessitates a combination of approaches (14).

NCDs are the leading cause of death in India (42%) in all regions, though it is highest in the southern states (15). Kerala, one of the south Indian states and the setting for this study, is in a unique position as it is the most advanced among Indian states in the epidemiologic and demographic transition (16). It has the highest prevalence of most NCDs, including diabetes and coronary heart disease and their risk factors (16, 17). It also stands out from the rest of India because of its high literacy, including high female literacy and health indicators that are comparable to many high-income countries (16).

Most of the initial NCD intervention studies conducted among the adult population in India have focused on diabetes and included diet as a component of lifestyle modification in a clinic or a community setting (18, 19). More recently, as part of an overall lifestyle modification program – with multiple strategies and activities covering individual and community empowerment, advocacy, and reorientation of health services – a community-based intervention for NCD risk factor reduction demonstrated only a modest, though statistically significant increase in the proportion of individuals consuming more than five servings per day of fruits and vegetables (FV) (women: from 3% at baseline to 5%, and men: from 5% at baseline to 9%) in spite of the very low baseline levels (20). Other intervention studies that have a dietary component have not yet published their results (21, 22).

The dietary component of the NCD prevention or lifestyle modification interventions in the Indian context has been restricted to counselling by nutritionists based on the National Institute of Nutrition's dietary guidelines for Indians (23) and the cultural adaptation has been limited to the inclusion of local foods on the menu (19, 20). A couple of studies have included culturally appropriate dietary education: addressing cultural barriers to dietary change (18, 22); use of local facilitators to deliver lifestyle classes (22); promoting local low-cost resources; and using cooking demonstrations, recipe competitions, and model meals (18). In general, most dietary guidelines are theoretical recommendations based on calorie requirements for different population groups (23). Some countries have attempted to translate their national guidelines for the benefit of the general population using nutrition labels, like ‘pick-the-tick’ or the ‘green keyhole’ in New Zealand and Sweden, respectively (24). Such strategies will work in contexts where consumption of ready-to-eat or processed foods exceeds that of home-cooked foods (25). In contrast, food preparation is an expected and central activity in Indian HHs, intrinsically linked to a woman's identity, and food-cultures differ widely even within the different states (7). Hence, a gap exists, not just in the lack of translation of available scientific knowledge in a format that is understandable and useful to different stakeholders, including the general population (14), but also in its process of implementation, which is important for sustainable behavioural and health outcomes.

## Rationale

Based on available evidence worldwide to decrease the population risk for major NCDs, World Health Organization (WHO) has urged member states to concentrate on four behavioural risk factors: diet, physical activity, alcohol and tobacco use (26). Nine major risk factors have been identified for myocardial infarction in Asian men and women: smoking, raised Apo-B to Apo-A1 ratio, history of hypertension, diabetes, abdominal obesity, psychosocial factors, daily inadequate consumption of FV, regular alcohol consumption, and inadequate physical activity (27). Five of these are directly related to diet.

We know that information or awareness alone is insufficient to modify behaviour (28). This calls for context-specific behavioural and policy interventions (1) that attempt to change population behaviour within the framework of other complex issues like culture, society, and family. The resource crunch, together with the high prevalence of NCDs and the high cost of accessing health care (16, 29), makes it imperative to focus more seriously on prevention. Preferably, all levels of prevention should be strengthened with more thrust at the initial levels as they have the potential for maximum impact at the population level. Hence, one of the challenges is to translate the available knowledge effectively into practical day-to-day reality that becomes an integral part of a HH's dietary practices. So far, existing knowledge has remained at the level of general dietary recommendations in many low- and middle-income settings including India.

Considering the paucity of related knowledge translation studies pertaining to the Indian context, we identified the need to design an effective model to transfer available scientific knowledge through a practical and community-based approach, focusing on changing unhealthy diet. The objective of this paper is to describe the process of designing the community-based Behavioural Intervention for Diet (BID) study with the aim to change dietary behaviour in Kerala based on local context and addressing local needs.

## Methods

The Intervention Mapping Protocol (30) was used as the basis for the planning and development of the BID study. The Methods section is described in terms of the study setting, the methodology adopted for the formative research, and the detailed study design of a cluster-randomized controlled trial (RCT) to test the effectiveness of a community-based dietary behavioural intervention.

### Study setting

Thiruvananthapuram (Trivandrum) district with a population of about 3.3 million inhabitants was chosen for two reasons: its human development index is similar to that of Kerala state, making it fairly representative of the state (16); and its proximity to a premier public health institution in the country for ease of monitoring. *Chirayinkeezhu taluk* is one of the four revenue divisions of Thiruvananthapuram district with a population of 550 thousand (about 130 thousand HHs). It is divided into four block *panchayats* which in turn consists of 22 *grama panchayats* (rural administrative unit) and two municipality areas (urban administrative unit). Each *grama panchayat* is further divided into 10–17 smaller administrative areas called wards.

In terms of dietary practices, coconut is a major ingredient of the Kerala diet in the form of both fresh, grated coconut and coconut oil and accounts for 80% of the fat intake (17). Fish is the other major food item, but the benefits are greatly reduced by the popular habit of deep-frying. A recent cross-sectional investigation of regional dietary patterns in Kerala (Thiruvananthapuram district) revealed a *pulses and rice* primary pattern characterised by mixed dishes mainly composed of pulses and fermented rice and a secondary *sweets and snacks* pattern characterised by sweet and fried savoury snacks (31). The other major trends include the increased consumption of animal source proteins (17, 32), both at home and during functions (7); high rice and low vegetable consumption (7, 17); increased frequency of eating (three meals and at least two snacks daily) (7); and the increased tendency to eat-out reflected in the rapid mushrooming of Gulf-style fast food restaurants (7).

### Formative research

Formative research was carried out to: 1) understand the social determinants of dietary structure, dietary choices, and decision making in HHs, particularly issues relating to access; 2) understand myths related to foods, particularly FV; 3) understand the feasibility and acceptability for the proposed study; and 4) gain insight into the kind of strategies that may be practical at community and HH level. Three focus group discussions (FGDs) and 17 in-depth interviews were conducted among residents of *Chirayinkeezhu taluk*. The participants were men and women aged between 24 and 75 years, and of different religions and socio-economic groups. An informal assessment of HH consumption and procurement of FV, salt, sugar, and oil was also carried out. Observations at market places and provision stores were also undertaken to understand the dynamics involved in the purchase of FV. For the quantitative verification of the prevalence of the behavioural risk factors, we used the results of two cross-sectional surveys done in the same population, namely WHO STEPs surveillance data (2003–05) (16) and a 5-year follow-up (2010) (Thirunavukkarasu S, unpublished results, 2010).

A conceptual model was constructed, from a combination of the trans-theoretical model of readiness-to-change (33), and two theories of social cognition, namely the Health Belief Model (34) and the Theory of Planned Behaviour (35), and modified after the formative research to incorporate ground realities. The conceptual model is composed of three parts: a) a three-stage change model consisting of pre-contemplation, intention, and action, which has been modified from the original five-stage trans-theoretical model (33); b) ‘impact factors’ that influence or act at various stages and; c) serial processes that enable progress from one stage to the other called ‘change processes’. In addition, there are modifiers, which are the societal and gender norms and three overarching influences, namely affordability, availability, and perceived needs and preferences.

Based on the formative research, a matrix on behaviour change objectives and what activities should be included in the programme was developed at three levels: individual, HH, and community (Tables 1 and 2).

**Table 1:** Individual level: behaviour change objectives with facilitating methods and strategies

Levels	Objectives	Role of the intervention	Methods and strategies^1^
Individual	1. To increase intake of fruits and vegetables to five (three vegetable and two fruit) servings daily	Increase awareness regarding diet and risk of NCDs and need for behaviour change Change intake behaviour to include more fruits and vegetables in the daily diet	Information booklet Counselling Substitution Visibility

	2. To decrease consumption of salt, sugar and oil in the daily diet	Identify and avoid high salt, sugar, fried food items or occasions for consuming such foods from the regular diet Change eating behaviour to avoid adding extra salt and sugar	Information booklet Counselling Substitution Visibility

	3. Increase self-efficacy at the individual level to make these changes	Increase confidence to bring about these changes by developing easy-to-use formulas and strategies Enable a positive environment at the HH and community level to make it acceptable and easy for an individual to choose a healthier food habit	Information booklet Counselling Telephonic reminder Awareness classes: adult & children Easy formula Substitution

NCD: non-communicable disease.

1 Details regarding the methods or strategies listed are available in the text of the article.

**Table 2:** Household and community level: behaviour change objectives with facilitating methods and strategies

Levels	Objectives	Role of the intervention	Methods and strategies^1^
Household	1. To enable a HH environment that would help any willing members make necessary changes	Increase collective awareness in HHs regarding diet and risk of NCDs and the need for behaviour change Help HHs measure the use of these items using simple HH measures and easy formulas	Information booklet Counselling Easy formula Substitution Visibility Staging of HHs Sequential stage-matching of strategies

	2. To increase procurement of fruits and vegetables	Help to identify opportunities to increase purchase or procurement of fruits and vegetables	Information booklet Counselling Substitution Local varieties Budget re-allocation

	3. To limit purchase or procurement of salt, sugar, oil and related food items	Understand recommended levels of intake for salt, sugar and oil and its relation to NCDs Identify decision points to reduce use of salt, sugar, oil during food preparation or purchase of related food items	Information booklet Counselling Substitution Budget re-allocation

	4. Increase self-efficacy at the HH level by improving their ability to make the necessary changes	Enable a positive environment at the HH and community level to make it acceptable and easy to change food procurement and preparation habits	Counselling Telephonic reminder Awareness classes: adult & children Budget re-allocation Sequential stage-matching of strategies

Community	To provide a supportive environment to enable HHs to make changes without fear of ridicule	Increase collective awareness at the community level regarding diet and risk of NCDs and the need for behaviour change	Awareness classes: adult and children

HH: household; NCD: non-communicable disease

1 Details regarding the methods or strategies listed are available in the text of the article.

### Final study design for the cluster RCT

#### Study objectives

We formulated our study objectives as follows:

Major. To test the effectiveness of a sequential stage-matched intervention strategy to increase the prevalence of adequate daily intake of FV (five servings = two Fruits + three Vegetables) by an absolute 20% from baseline in the intervention arm over a one-year intervention period.

Minor. 1) To increase HH procurement of FV by a minimum of 20% and decrease HH consumption of salt, sugar, and oil by a minimum of 10%; 2) To assess whether sequential stage-matched interventions can assist households to progress through the different stages of change at subsequent follow-up points; and 3) To assess barriers and facilitating factors for dietary behaviour change in terms of change processes with particular reference to gender and SES.

### Study population

#### Sample size

With an existing prevalence of 40% appropriate daily intake of FV (five servings) (Thirunavukkarasu S, unpublished results, 2010) and assuming a 20% improvement in the intervention arm, with a power of 80% and an alpha error of 0.05, the minimum sample size required to compare two groups was calculated to be 98 in each group (36). As intra-class cluster correlation coefficient was not available for the outcome of interest, we incorporated a design effect of two. Hence, the minimum required sample size at baseline would be 400 (200 intervention and 200 control). To allow an attrition of 20%, and to ensure these numbers at end-line, considering a risk factor prevalence up to 50% (16) and individuals in the required age group of 25–45 years, we screened 1237 HHs and recruited 479 individuals (intervention: 240, and control: 239). We developed a cluster RCT with fixed number of clusters (*ayalkootams*: 30 in each arm) and variable cluster size ranging from 6 to 11 (average cluster size: 8.5).

A HH was found to be eligible for recruitment into the study if it fulfilled all of the following inclusion criteria:

1. Belonged to the middle SES.
2. Had adult males or females 25–45 years of age.
3. Had resided in selected administrative areas (panchayats) of rural *Chirayinkeezhu* *taluk* for at least 6 months prior to the screening survey.

The SES of HHs were identified for 2011 based on HH monthly per capita expenditure using the 2004–2005 poverty line (37) and the corresponding affluence line (38) for rural Kerala, after adjusting for inflation.

One male or female individual aged 25–45 years was selected in each HH after exclusions based on the following criteria: 1) Took two or more meals away from home on most weekdays; 2) Had definite plans of migration during the study period; 3) Was pregnant; or 4) Had self-reported diabetes, hypertension, deranged lipids on medical treatment, and mental or other serious illness.

#### Sampling procedure

**Fig. 1:**
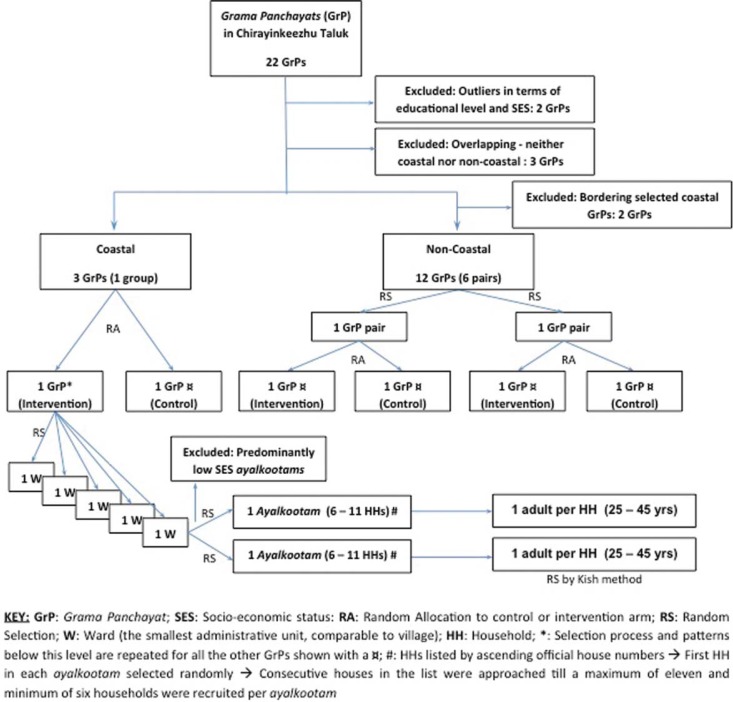
Randomization and selection of intervention and control *panchayats*.

We used a multistage stratified cluster RCT design whereby six *grama panchayats* were finally selected for the study (three in the intervention and three in the control arm) (Fig. 1). At the outset, three criteria were used for pairing of *panchayats* to ensure uniform distribution of key population characteristics and confounders in the control and intervention groups: 1) coastal and non-coastal areas were paired separately; 2) similar proportion of middle-income HHs; and 3) *panchayats* belonging to one pair or group should not share a border to prevent spill-over effect.

All randomizations were done using an online random number generator from Random.org. After pairing the *panchayats*, one random number was selected which was allocated to the intervention arm and the remaining *panchayat* automatically became part of the control arm. In the group with three *panchayats*, two of them were randomly selected of which the first random number was allocated to the intervention arm and the second to the control arm. Further to this, five wards per *panchayat* and two *ayalkootams* per ward were randomly selected, before reaching the household level. At the household level, if more than one individual fulfilled the inclusion criteria, then Kish methodology was used (39). The age group 25–45 years was targeted due to the high prevalence of migration in the younger age group and of NCDs among the older age group.

Rural Kerala has neighbourhood groups organized through the *Kudumbasree* (women oriented, community based, and organized by the State Poverty Eradication Mission of Government of Kerala) known as *ayalkoottams*. These are present in each ward and composed of women members from the community. Approximately 80% of low- and middle-income HHs (at least one person from each HH) are enrolled in these units as registered members. The clusters in this study are the *ayalkootams*. Predominantly, low SES *ayalkootams* (verified through *Kudumbasree* registers) were excluded, and the ones remaining were used as the basis for random selection of clusters. All HHs falling within geographic limits of the selected *ayalkoottams* were listed and screened before inclusion and exclusion criteria were applied. The final selection of HHs was proportionate to the numbers screened per *ayalkootam* and ranged from six to eleven HHs.

### Intervention components

**Fig. 2:**
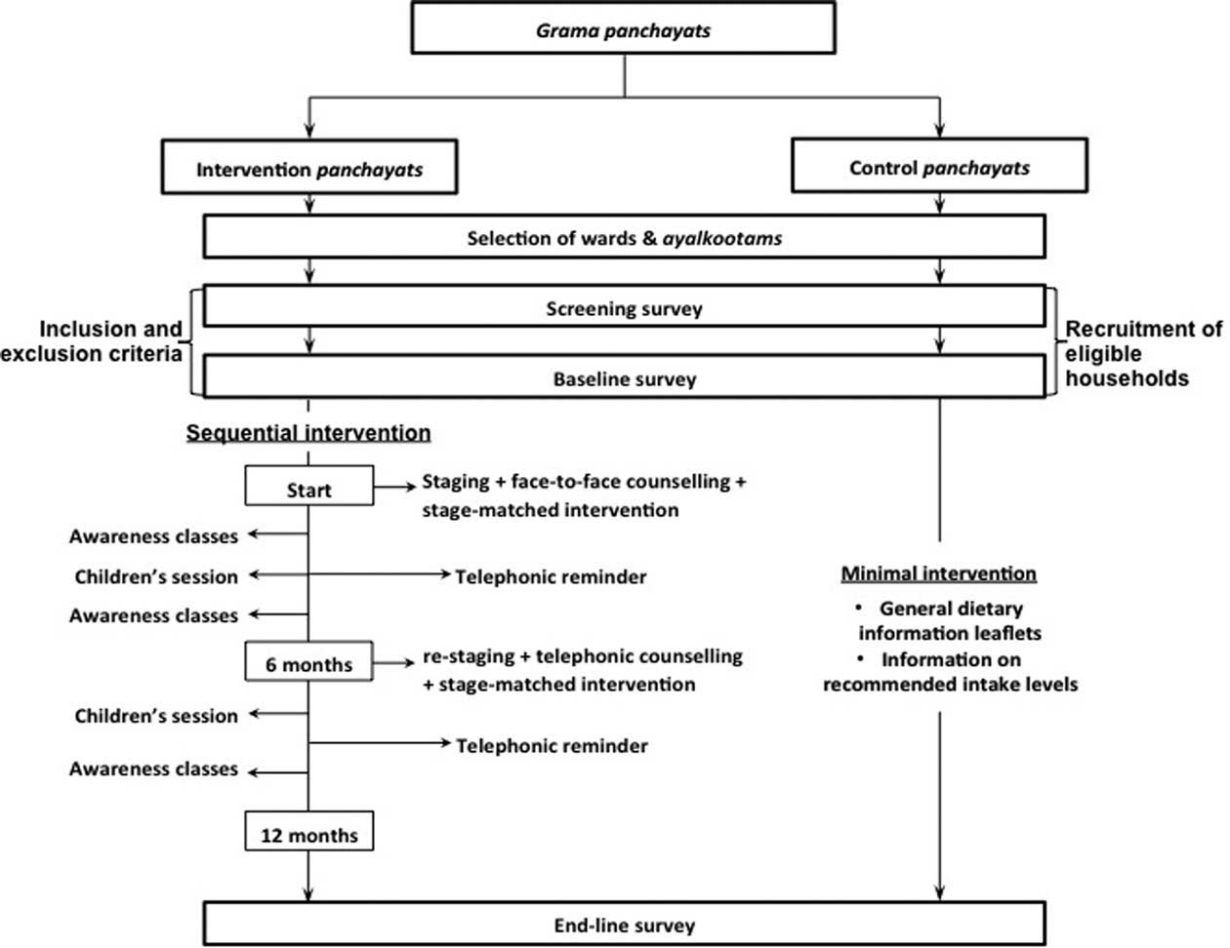
Detailed study plan showing intervention components.

The intervention was applied at the HH and *ayalkootam* level. Intervention strategies at the individual level comprised of participation in the initial face-to-face counselling and the community-based awareness classes (Fig. 2). The strategies developed were applied in a staggered manner in the intervention areas, while the control areas received minimal intervention in the form of information on recommended levels of intake of the five dietary components, and general dietary information leaflets at the end of the baseline survey. The intervention strategies are outlined below as general strategies that were applicable to all HHs, irrespective of stage of change, and stage-matched strategies that were applied based on a HH's stage of change.

#### General strategies (i–iv)

- Counselling: Counselling was performed face-to-face at the beginning of the intervention, and via telephone at six months. The contents of each counselling session were however tailored to the specific stage-of-change to help HH members choose appropriate stage-matched strategies. Each counselling session was accompanied by a simultaneous home visit, when a community volunteer demonstrated the most effective method of applying the chosen strategy.
- Telephonic reminder: This was done at two different time points by the community volunteers or study coordinator who called the female head of the HH and if possible the randomly selected individual.
- Awareness classes: Three adult awareness classes were conducted per *ayalkootam* facilitated by a trained resource person each addressing a different theme: 1) Diet and NCDs; 2) Colour way to health; 3) Let us protect our family's health. Separate flipcharts were prepared for each theme. Two children's awareness classes were conducted per *ayalkootam*, addressing the first two adult session themes, with a specially developed card game for each session. The sessions were open to all interested community members (not just recruited HHs) to foster a favourable environment that would encourage healthy dietary change among HHs in the locality.
- Sequential stage-matching: HHs were assessed regarding progress through the three stages of change by re-staging during month 6, with the opportunity to choose additional strategies matched to the new stages. Initially, re-staging, telephonic counselling, and home visits were planned for two intermediate time points, month 4 and 8. However, due to practical reasons, this was reduced to one intermediate time-point at month 6.

#### Stage-matched strategies (a–e)

- *Easy Formula*: HHs were equipped with a measurement kit, to enable them to measure their use of five dietary components on a regular basis. Based on felt need, we also translated daily consumption targets (individual and HH) for FV, salt, sugar, and oil to locally available HH measures provided through the kit. As per caloric requirements in the guidelines (23), we calculated recommended levels of free sugars and cooking fat for a reference family identified from the latest available census data pertaining to *Chirayinkeezhu taluk* (40).
- *Substitution*: Substituting unhealthy food with healthier options: 1) fried snacks with fruits; 2) ready-to-eat or fried snacks with home-cooked or steamed snacks respectively; and 3) increasing the colour of the lunch plate by substituting a portion of the rice with raw or cooked vegetables.
- *Visibility*: Increasing the display of FV on the dining table or easily accessible locations in the house and decreasing the display and access for jams, pickles, and fried items.
- *Local varieties*: Encouraging the use of locally available FV, during house visits where the community volunteers helped the HHs to record the varieties they would be willing to try in their information booklet.
- *Budget re-allocation*: Teaching HHs how to reallocate their budgets to increase the money available to purchase FV without exceeding overall food budget.

The strategies (a–e) were matched to specific stages of change, based on perceived difficulty derived from our formative research:

1. Pre-contemplation stage: only general strategies.
2. Intention stage: general + stage-matched strategies (a–c).
3. Action stage: general + stage-matched strategies (a–e).

The counsellors helped the HH members to identify the strategies which they felt comfortable to try for the following 6 months from the strategies that were available to them, while the community volunteers helped them work out the details of the chosen strategies based on a pre-defined steps aided by specific tools.

#### Intervention tools

1) Information booklet for the intervention HHs outlining the strategies and general dietary information leaflets for intervention and control HHs; 2) Household measurement kit which included: an oil jar to keep track of monthly HH consumption, a small graduated cup to measure oil for daily use tailored to each HH size, a serving spoon (*thavi*) to measure one serving of cooked vegetables, a fruit basket to display fruits or vegetables, and salt and sugar containers with 5 g scoops. Each item was labelled with catchy slogans and the recommended consumption level (per person per day) in the local language, Malayalam; 3) Flip charts outlining the content of the adult awareness classes and; 4) Two card games for teaching children about the concept of ‘plus’ (healthy) and ‘minus’ (unhealthy) foods and the importance of ‘colours of health’ with respect to fruit and vegetable consumption. The information booklet, flip charts for the adults’ awareness classes, and card games for the children's awareness classes were designed by the first author (MD), pre-tested on appropriate but substitute target populations, and modified before training of resource persons. The information booklet was verified for content by three of the co-authors and an independent expert. The flip charts and card games were verified by two of the co-authors.

### Data collection

#### Tools

Tools were developed for: 1) screening; 2) data collection at the individual and HH level; 3) process documentation to keep track of and evaluate the various processes and strategies involved in the intervention; and 4) template counselling sheets specific to stage of change of the HH to guide counsellors during face-to-face and telephonic counselling. A new tool had to be developed and validated to identify the stage-of-change at HH level. All data collection tools were piloted three times before the start of the study.

The HH-level data collection tool included demographic information, food procurement and preparation habits and a 2-week procurement diary for FV, while individual-level data included demographic data, dietary intake habits, two 24-h dietary intakes (weekend + weekday), and a five-option evaluation of strategies at end-line. The 24-h recalls incorporated the multiple pass method (41): an initial listing of foods; followed by progressive addition of consumption amounts using pre-tested serving spoons to assess consumption of different food items; and incorporated a food map to allow recall of the place, occasion, and company during each meal to aid in remembering any forgotten meals. Salt, sugar, and oil intake for the HHs were assessed in terms of monthly consumption for the HH based on the amount procured and the duration of use. For sugars and oils, we collected data for different types of sugars, including jaggery, and different types of oils or fats, including hydrogenated oils, used in the HHs.

Process documentation tools were developed for each of the intermediate points, namely face-to-face and telephonic counselling, adults and children's awareness classes and telephone reminders. These enabled the documentation of the number and type of participants, problems faced and any other general comments.

#### Process

Quantitative data collection started with a screening after which the inclusion and exclusion criteria were applied followed by baseline data collection from the recruited HHs at the individual and HH level. All data except the four-point evaluation were collected at baseline and repeated after one-year. The qualitative part of data collection consists mainly of the in-depth interviews and FGDs at the end of one-year, which addresses barriers and facilitators for behaviour change based on change processes, factors that favour sustenance of behaviour change over longer periods and understanding gender and socio-economic dimensions of dietary behaviour change process.

#### Monitoring

Process documentation tools were used to monitor all key activities. Using the tools, we tracked and ensured that the different strategies at different time points were delivered to each HH. For the community awareness sessions, the attendance was recorded, and attendance by any members of the recruited HHs was specifically marked. In addition, quality checks were put in place to ensure that activities were carried out. For example, the community volunteer scheduled the counselling and house visits, not the team performing the activity. Moreover, the coordinator carried out random telephone checks by calling the HHs directly to enquire about a particular scheduled activity.

#### Roles and responsibilities of team members

Data collection and intervention delivery were carried out by 15 teams (five teams per *panchayat*) composed of two community volunteers, one each from the *Kerala Mahila Samakhya Society* (Kerala chapter of the ‘Education for women's equality’ programme launched by the Government of India) and the *Kudumbasree*, where at least one member was a local resident with good knowledge of the selected HHs and their members, key stakeholders, and local politics. All community volunteers had a minimum of 10 years of schooling, had been involved in data collection for health-related surveys in the past, and had received training on data collection tools and methods.

Four teams in each *panchayat* participated in the general data collection, while all volunteers were involved in the organization of the community awareness sessions in their respective areas. One team per *panchayat* was responsible for administering staging questionnaires in a *panchayat* other than their own to minimise socially desirable responses to the staging questions. The same teams also conducted house visits to explain the stage-based strategies in their own *panchayat*, as they only helped the HHs to work out the details of the strategies that they had chosen with the counsellors. These three teams (one from each *panchayat*) were excluded from the general data collection to prevent inducing social desirability bias. Three counsellors, who were outsiders to the community, conducted the counselling and community awareness sessions. Similarly, the study coordinator, who was also an outsider, conducted the overall monitoring and quality checks.

### Data analysis

Intention-to-treat analysis will be used to evaluate the effectiveness of the intervention at two levels: HH and individual level, with adjustments for cluster design effects. Our primary outcome measures will include change in FV intake at individual level and FV procured at HH level over the study period. The secondary outcome measures include change in salt, sugar, and oil consumed at the HH level and proportion of HHs progressing across the stages of change at 6 and 12 months. Barriers and facilitators for behaviour change will be analysed using content analysis of transcripts of interviews and FGDs conducted at the end of the intervention.

### Ethical considerations

This study was conducted according to the guidelines laid down by the Indian Council of Medical Research and all procedures involving the study participants were approved by the Institutional Ethics Committee (IEC) of Sree Chitra Tirunal Institute for Medical Sciences and Technology, Thiruvananthapuram, Kerala, India (SCT/IEC-357/MAY-2011; Date: 11/06/2011). Participants were recruited into the study only after written informed consent. The study was also registered prospectively under the Clinical Trial Registry of India (CTRI/2011/06/001839; Date: 28/06/2011) (42).

## Discussion

### Context-specific factors influencing the study design

Based on our formative research, we identified a number of context-specific factors that influenced the study design. First, the need to address the *HH as the decision unit* for food-related issues in rural Kerala, which has also been implied elsewhere (7). Hence, the stage of change had to be identified for the HH rather than the individual. We therefore developed and validated a staging tool for HHs, which, to the best of our knowledge, has not been attempted so far in any behavioural intervention study. We staged HHs to one of three stages and also devised an on-going mechanism to facilitate progress through the stages. Hence, the interventions were delivered according to the stage of change in a sequential manner. Stage-matched strategies have been used in other studies (43, 44), but as far as we have found, this is the first study to attempt sequential stage-matching.

Second, *coastal and non-coastal areas* differed in terms of foods consumed and access to FV markets. The study design had to ensure representation of both areas in the intervention and control arms; hence, stratification and pairing was carried out. Third, *low SES HHs* were found to be almost completely unresponsive and would require completely different strategies and some form of subsidy, which was beyond the scope of this study. Hence, the range of the middle class monthly per capita expenditure was calculated, and it was decided to exclude all families who fell on either sides of this range. Since Kerala has a large middle-income group (85%) (38), we still included the majority of the population. Moreover, the intervention clusters (*ayalkootams*) needed to be more homogenous. Otherwise, we would have required multiple groups of interventions targeting different SES levels.

Fourth, we identified a need to address *salt, sugar, and oil consumption in addition to FV intake*. The BID study was initially proposed as an intervention to increase FV intake, which was inadequate as per previous study findings (16, Thirunavukkarasu S, unpublished results, 2010). During the informal assessment carried out as part of the formative research, HH consumption of salt, sugar and oil was found to be high. Since the majority of salt and oil consumption in transition economies is through HH cooking rather than processed or ready-to-eat foods (25), it was decided to focus our intervention on five dietary components, namely fruits, vegetables, salt, sugar, and oil. Consumption of coconut products was also found to be high. This was addressed through the community awareness classes, focusing on reducing the use of coconut meat and using a mix of cooking oils during food preparation.

Fifth, we also identified a need for *easy measurement formulas*, expressed by participants to understand the recommended levels for each of the five-targeted dietary components, in terms of per person per day measures. A HH measurement kit was assembled to partly address this gap. While recommended daily levels of intake were available for FV and salt, this was not the case for sugar (free sugar) and fats. Most dietary guidelines, including the one for Indians, advise visible fats as not exceeding 10% of total energy intake (23). Therefore, relevant dietary recommendations were adjusted for local dietary practices (e.g. use of coconut), converted to per person per day levels, and translated to easy-to-use HH formulas using locally available measuring spoons.

Finally, we also realised that *promoting men's participation* would be important. Otherwise, we ran the risk of the whole project being labelled as a ‘women's issue’ with very little or negligible male participation if all the study personnel were women. Spouse's preferences were found to be a key consideration in the dietary decision-making process. Hence, we decided to recruit men for the positions of counsellors and study co-ordinators, to encourage more male participation within the HHs and communities.

### Community as a driver and supporter of behaviour change

Communities, peers and families have often been used as ‘change agents’ for many community-based interventions (45). That they exert varying degrees of influence on individuals is well documented, which is precisely the reason why they work as ‘change agents’. In complex multicultural societies like India and Kerala, the approval or support of these ‘change agents’ is often crucial. It was not enough to increase awareness and educate the target HHs alone, we also had to devise strategies to enable communities to be more receptive, so that these changes became possible and even expected. A community or family that shuns or ridicules the proposed changes can cause serious setbacks. Hence, the communities and other HH members, particularly children, were also included as part of the broader intervention package. Due to the high prevalence of NCDs (16), most people were partly aware of this reality and even concerned, particularly for their children, which we identified as a powerful motivator.

### Sustainability as a key issue

Finally, the study design had to incorporate elements, which would make long-term sustainability possible. To this end, the study made maximum use of existing infrastructure in the form of community volunteers, self-help group networks, and existing administrative divisions. The use of community volunteers is expected to minimise both non-response as well as dropout rates as they are living in these communities and know most of the study participants. Currently, many of them are also employed part-time through the Mahatma Gandhi National Rural Employment Guarantee Scheme, where they are paid daily rates for unskilled jobs. For a country, like India, that is struggling with unemployment on the one hand and lack of adequate health manpower on the other, this may represent a tremendous opportunity to link the two. This study has the potential to demonstrate that community volunteers, particularly in a highly literate state like Kerala, can deliver complex interventions, provided they receive adequate training and that knowledge has been appropriately translated into a form that can be used by them. Training of community volunteers and constant reinforcement of this knowledge could enhance the basic knowledge and practice levels in their communities, thus improving the long-term sustainability of such interventions.

## Limitations and strengths

We have not included the low and high SES. Even though middle class in Kerala comprises 85% of the population (38), this would still affect the generalizability of the results.

In 2008, the pilot phase of the National Programme for prevention and control of Cancer, Diabetes, Cardiovascular diseases and Stroke (NPCDCS) was launched in seven states with one district each, including Thiruvananthapuram district of Kerala state. The recruitment for the BID study started in August 2011. Awareness sessions aired on the state-run radio and television channels address all NCD risk factors, including diet, in general, and may have positively influenced the HHs to seriously participate in our intervention. However, it is likely to impact both the intervention and control groups equally.

This study was based on extensive formative research and addressed the felt needs of the community, which is a definite strength. The strict randomization process that was followed right down to the individual level minimises bias to a large extent. In terms of strategies developed to achieve the objectives, one of our major strengths is that none of the strategies try to achieve the objective through increasing the HH food budget; rather it is through re-allocation, use of nutritious local varieties, and the substitution of unhealthy food choices. Similarly, the inclusion of community volunteers, who have access to both the HHs and the local administration, had a positive influence on the acceptability of the intervention.

## Conclusion

It was possible to design a cluster-randomized intervention trial for dietary behaviour change, taking into account the socio-cultural context, the felt needs of the community, and the ground realities associated with existing dietary procurement, preparation, and consumption patterns. Some innovative tools and strategies were developed to assess a HH's readiness-to-change behaviour, increase awareness at HH and community level, and make the practical side of behaviour change easier through a HH measurement kit and easy formulas. The study design has also addressed some of the key issues raised during formative research, particularly the central role of the HH as the decision unit, and has tried to increase the long-term sustainability through the use of existing local and administrative networks and community volunteers.
